# Brood-Derived Fat Extracts from *Apis mellifera* as Sustainable Alternatives to Beeswax in Topical Nanostructured Lipid Carriers

**DOI:** 10.3390/biology15060472

**Published:** 2026-03-14

**Authors:** Piyathida Samianpet, Suvimol Somwongin, Rewat Phongphisutthinant, Supakit Chaipoot, Pairote Wiriyacharee, Singkome Tima, Songyot Anuchapreeda, Saranya Juntrapirom, Watchara Kanjanakawinkul, Thomas Rades, Wantida Chaiyana

**Affiliations:** 1 Department of Pharmaceutical Sciences, Faculty of Pharmacy, Chiang Mai University, Chiang Mai 50200, Thailand; piyathida_sami@cmu.ac.th (P.S.); suvimol.s@cmu.ac.th (S.S.); 2 Research Center of Deep Technology in Beekeeping and Bee Products for Sustainable Development Goals: SMART BEE SDGs, Chiang Mai University, Chiang Mai 50200, Thailand; 3 Multidisciplinary Research Institute, Chiang Mai University, Chiang Mai 50200, Thailand; rewat.p@cmu.ac.th (R.P.); supakit.ch@cmu.ac.th (S.C.); 4 Research Center of Microbial Diversity and Sustainable Utilization, Faculty of Science, Chiang Mai University, Chiang Mai 50200, Thailand; pairote.w@cmu.ac.th; 5 Faculty of Agro-Industry, Chiang Mai University, Chiang Mai 50100, Thailand; 6 Division of Clinical Microscopy, Department of Medical Technology, Faculty of Associated Medical Sciences, Chiang Mai University, Chiang Mai 50200, Thailand; singkome.tima@cmu.ac.th (S.T.); songyot.anuch@cmu.ac.th (S.A.); 7 Center of Excellence in Pharmaceutical Nanotechnology, Faculty of Pharmacy, Chiang Mai University, Chiang Mai 50200, Thailand; 8 Chulabhorn Royal Pharmaceutical Manufacturing Facilities by Chulabhorn Royal Academy, Chon Buri 20180, Thailand; saranya.jun@cra.ac.th (S.J.); watchara.kan@cra.ac.th (W.K.); 9 Department of Pharmacy, Faculty of Health and Medical Sciences, University of Copenhagen, Universitetsparken 2, 2100 Copenhagen, Denmark; thomas.rades@sund.ku.dk; 10 Multidisciplinary and Interdisciplinary School, Chiang Mai University, Chiang Mai 50200, Thailand

**Keywords:** *Apis mellifera* brood, honeybees, nanotechnology, nanostructured lipid carriers (NLCs), sustainable use of natural resource, brood-derived lipids, lipid extraction, alternative lipid sources, innovative cosmetic ingredients

## Abstract

This study evaluated *Apis mellifera* brood fat extracts as a sustainable alternative to beeswax for anti-inflammatory topical delivery, including their formulation into nanostructured lipid carriers (NLCs). Brood fat was extracted using acetone, ethyl acetate (EA), and hexane, and the resulting extracts were characterized for fatty acid composition and physicochemical properties. Safety was assessed using the hen’s egg chorioallantoic membrane test and cytotoxicity testing in RAW 264.7 macrophages. Anti-inflammatory activity was assessed by inhibition of lipopolysaccharide-induced interleukin-6 (IL-6) and tumor necrosis factor-alpha (TNF-α) production. The most suitable extract was formulated into NLCs using sugar squalane as liquid lipid, and the effects of lipid ratio and preparation method were investigated. The results showed that the ethyl acetate extract had the highest yield. Compared with beeswax, all fat extracts exhibited a favorable oleic acid–rich fatty acid profile with comparable crystallinity and thermal behavior, while showing significantly enhanced anti-inflammatory activity (*p* < 0.05). All extracts and their NLCs were non-irritating and non-cytotoxic. Ethyl acetate extract-based NLCs exhibited favorable particle sizes (72.1 ± 0.3 nm) and narrow polydispersity (0.14 ± 0.00), with high-pressure homogenization producing smaller particles compared to probe sonication without affecting IL-6 or TNF-α inhibition. Therefore, *A. mellifera* brood fat extract is a sustainable anti-inflammatory lipid source with strong potential as an alternative to beeswax in topical nano-formulations.

## Simple Summary

This study investigated a new and more sustainable approach to developing ingredients for skin care products with anti-inflammatory properties. Beeswax, a natural substance produced by honeybees, is commonly used in many skin care products to help deliver active ingredients to the skin. However, relying only on beeswax may not be the most sustainable option. The researchers therefore explored whether fat from honey bee brood, which are developing young bees, could be used as an alternative. The fat was extracted using different solvents and then tested to examine its composition, safety, and ability to reduce inflammation. The results showed that extraction using ethyl acetate produced the highest amount of useful fat. The brood fat contained beneficial fatty acids that support skin health and showed stronger anti-inflammatory effects than beeswax. Safety tests confirmed that the fat did not irritate tissue or damage cells. The researchers also produced very small fat particles designed to deliver active ingredients through the skin more effectively. These particles were stable, uniform, and maintained their anti-inflammatory activity. In conclusion, the findings suggest that honey bee brood fat could be a safe, effective, and environmentally friendly alternative to beeswax for improved skin care preparations.

## 1. Introduction

Beeswax is one of the most widely used natural lipids in cosmetic and pharmaceutical formulations due to its biocompatibility, film-forming ability, and stabilizing properties [1,2]. It is commonly employed as a stiffening agent, emollient, and structural lipid in topical products such as creams, ointments, and lipid-based delivery systems [3]. Despite its long-standing use, increasing demand for natural and sustainable ingredients has raised concerns regarding the long-term availability, functional limitations, and sustainability of beeswax as a primary lipid resource [4,5,6]. Beeswax production is inherently dependent on *Apis mellifera* colony productivity and environmental conditions [7,8]. Its supply is constrained by seasonal variation, hive health, and competing industrial demands, including cosmetics, pharmaceuticals, food coatings, and candle manufacturing [9]. Moreover, global challenges such as climate change, pesticide exposure, and colony collapse have contributed to declining *A. mellifera* populations, indirectly affecting beeswax availability and price stability [9,10]. In addition, beeswax is frequently adulterated with paraffin or synthetic waxes due to supply shortages, raising concerns over quality control, safety, and reproducibility in high-value formulations [11]. These limitations highlight the need to explore alternative lipid sources that are renewable, scalable, and functionally suitable for modern formulation technologies.

From a formulation perspective, beeswax is characterized by a high content of long-chain saturated fatty acids and wax esters, resulting in a rigid and highly crystalline structure [12]. While this property provides mechanical strength, it presents challenges for advanced lipid-based delivery systems such as nanostructured lipid carriers (NLCs), as high crystallinity can limit drug loading capacity, reduce formulation flexibility, and promote drug expulsion during storage [13,14]. As cosmetic and pharmaceutical research increasingly shifts toward nanotechnology-based delivery platforms, there is growing demand for lipid materials with reduced crystallinity, improved molecular flexibility, and enhanced compatibility with nanoscale systems [15,16]. To address these challenges, the identification of new sustainable lipid resources has become increasingly important. Ideally, such lipids should be biodegradable, non-toxic, skin-compatible, and suitable for incorporation into nanocarrier systems [17]. In this context, insect-derived lipids have attracted growing attention as underutilized but promising natural resources [18].

Insects are characterized by high lipid content, rapid renewability, and low environmental impact, making them attractive candidates within circular bioeconomy and sustainability frameworks [19,20,21]. *Apis mellifera* brood, which comprises the immature larval and pupal stages of worker and drone *A. mellifera* reared in wax comb cells [22], represents a particularly promising insect-derived lipid source. During routine beekeeping practices, especially royal jelly production, substantial amounts of brood are removed and treated as low-value by-products [23]. Traditionally, *A. mellifera* brood has been consumed as food in several regions of Africa and Asia due to its rich nutritional profile [24]. Recent studies have further demonstrated that proteins and protein hydrolysates derived from *A. mellifera* brood exhibit antioxidant, anti-aging, and low-irritation properties, supporting their potential use in cosmeceutical applications [25]. In contrast, the lipid fraction of *A. mellifera* brood, which is abundant and generated as a by-product during protein extraction, has received comparatively little attention. The lipid composition of *A. mellifera* brood fat is dominated by biologically relevant fatty acids, including oleic acid, palmitic acid, stearic acid, and linolenic acid [24]. Previous studies have reported that the lipid extracts of *A. mellifera* drone larvae exhibit in vitro anti-inflammatory effects by decreasing the mRNA expression of proinflammatory cytokines, including interleukin-6 and 10 (IL-6 and 10), inducible nitric oxide synthase (iNOS), and cyclooxygenase-2 (COX-2) [26]. Hence, it is worth investigating the extraction and utilization of the fat from the *A. mellifera* brood as an alternative active ingredient in cosmetic applications.

In addition to its biological activity, the formulation of lipid nanoparticles is worth investigating to further explore the application potential of *A. mellifera* brood, as fats are widely used in pharmaceutical and cosmeceutical products as emollients, stiffening agents, and carriers to improve skin appearance and hydration [27,28]. Lipid-based nanodelivery systems, particularly solid lipid nanoparticles (SLNs) and NLCs, have gained extensive use in topical formulations due to their ability to enhance stability, bioavailability, and skin penetration of active ingredients [29,30]. However, SLNs often suffer from drug leakage caused by their highly ordered crystalline lipid matrix, which compromises formulation stability [31]. To overcome this limitation, NLCs were developed by incorporating liquid lipids into solid lipids, reducing crystallinity and improving drug loading capacity and stability [32,33,34]. In NLCs, the imperfect lipid matrix allows controlled release of active compounds and improved formulation performance [35]. Therefore, biodegradable and skin-compatible lipids, such as *A. mellifera* brood fat extracts, represent promising candidates for the development of bioactive NLCs for topical applications.

Beyond formulation advantages, the utilization of brood-derived fat offers significant sustainability benefits. Valorizing *A. mellifera* brood fat aligns with waste reduction and circular bioeconomy principles by transforming an underutilized beekeeping by-product into a high-value functional ingredient. This approach not only reduces reliance on conventional beeswax but also provides additional economic opportunities for small- and medium-scale beekeepers. Therefore, the present study aimed to investigate suitable extraction methods for obtaining fat from *A. mellifera* brood and to evaluate its physicochemical properties, safety, and anti-inflammatory activity in comparison with beeswax. In addition, the most suitable brood fat extract was incorporated into NLCs, and the effects of formulation parameters and preparation methods on NLC characteristics were assessed. The advantages of *A. mellifera* brood fat–based NLCs were evaluated in terms of safety and anti-inflammatory activity in comparison with beeswax–based NLCs.

## 2. Materials and Methods

### 2.1. A. mellifera Broods Materials

**Figure 1:**
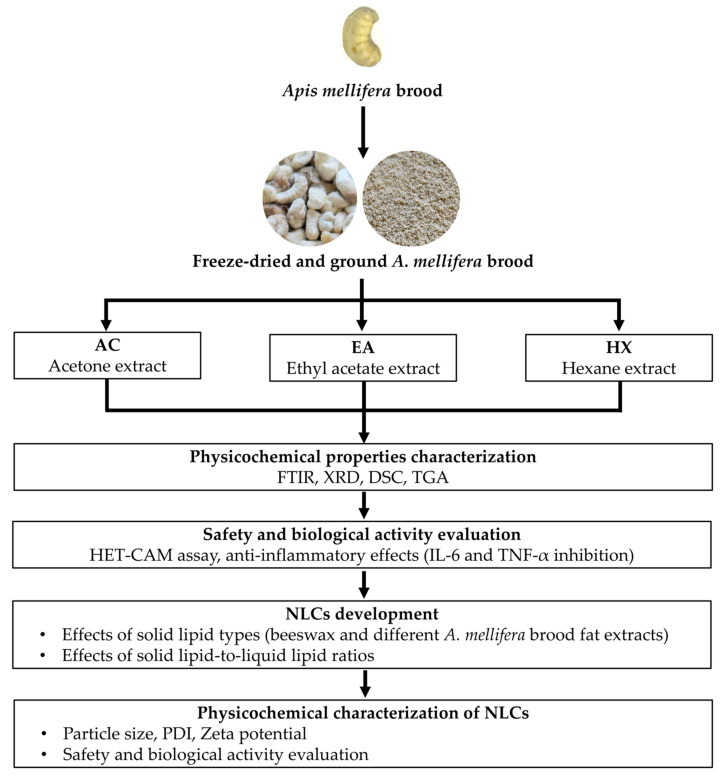
Schematic overview of the extraction, characterization, biological evaluation, and nanostructured lipid carriers (NLCs) formulation of *A. mellifera* brood fat extracts.

*A. mellifera* broods, purchased as edible commodities from a local market in Chiang Mai, Thailand, were taxonomically identified by Dr. Bajaree Chuttong, Meliponini and Apini Research Laboratory, Department of Entomology and Plant Pathology, Faculty of Agriculture, Chiang Mai University. The *A. mellifera* broods were categorized into their respective developmental stages, including the prepupa and pupa, based on their external morphology and shapes. The broods were subsequently subjected to freeze-drying using a LyoQuest laboratory freeze dryer (Telstar, Terrassa, Spain). Following lyophilization, the freeze-dried *A. mellifera* broods were pulverized into fine powders using a blender (HR2115 model, Phillip, Eindhoven, The Netherlands) and stored in tightly sealed containers at room temperature until further evaluation as shown in Figure 1.

### 2.2. Chemical Materials

Commercially available natural white beeswax (cosmetic grade, super-refined quality) was obtained from Chanjao Longevity Co., Ltd. (Bangkok, Thailand). Acetone (C_3_H_6_O), hexane (C_6_H_14_), ethyl acetate (C_4_H_8_O_2_), and deionized (DI) water were purchased from RCI Labscan Co., Ltd. (Bangkok, Thailand). Polysorbate 80 (Tween^®^ 80) and sorbitan oleate (Span^®^ 80) were purchased from Loba Chemie Pvt. Ltd. (Mumbai, India). Dulbecco modified eagle medium (DMEM), dulbecco’s phosphate-buffered saline (DPBS), isopropyl myristate (IPM), L-glutamine, penicillin/streptomycin, trypan blue dye solution, fetal bovine serum (FBS), 10 mM dexamethasone, and trypsin were purchased from Gibco (Waltham, MA, USA). (3-(4,5-Dimethylthiazol-2-yl)-2,5-Diphenyltetrazolium Bromide) (MTT) dye, sodium chloride (NaCl), dimethyl sulfoxide (DMSO), lipopolysaccharides (LPS), and sodium lauryl sulfate (SLS) were purchased from Sigma-Aldrich (St. Louis, MO, USA). Sugar squalane was purchased from Namsiang (Chiangmai, Thailand). Additionally, mouse tumor necrosis factor-alpha (TNF-α) and interleukin-6 (IL-6) uncoated enzyme-linked immunosorbent assay (ELISA) kits were purchased from Invitrogen (Grand Island, NY, USA).

### 2.3. Preparation of Fat Extracts from A. mellifera Broods by Solvent Extraction

The dried powder of *A. mellifera* brood was divided into separate batches and subjected to individual solvent extraction using three distinct solvents, including acetone (AC), ethyl acetate (EA), and hexane (HX). Extractions were carried out at a weight-to-volume ratio of 1:5 (*w*/*v*). Each mixture was shaken on an orbital shaker (Innova^TM^ 2100 Eppendorf, Hamburg, Germany) at 500 rpm for 24 h at room temperature. The solvent was collected after filtration through a Whatman^®^ No. 1 filter paper (Merck KGaA, Darmstadt, Germany), and the resulting residue was re-extracted two additional times with fresh solvent (a total of three cycles). Finally, the filtrates from the three extraction cycles were pooled, and the solvent was removed under reduced pressure using a rotary evaporator (Büchi Labortechnik GmbH, Essen, Germany) until dryness. The extraction yield was determined using the following equation:

Extraction yield (%) = A/B × 100(1)

where A is an amount of the *A. mellifera* brood fat extract, and B is the weight of dried *A. mellifera* brood powder. Each extraction was performed independently in triplicate. The extracts were stored at 4 °C until use.

### 2.4. Fatty Acid Profile Determination of A. mellifera Brood Fat Extracts by Gas Chromatography-Flame Ionization Detector (GC-FID)

All *A. mellifera* brood fat extracts were investigated for their fatty acid compositions by GC-FID. The analysis was conducted following the in-house method of Halal Science Center, Chulalongkorn University (Bangkok, Thailand), based on the Halal GMP/HACCP and Halal-QHS/ISO 22000 standards [36]. Briefly, the lipid part of the sample was extracted by liquid–liquid extraction method using 2:1 (*v*/*v*) dichloromethane-methanol as a solvent. To generate fatty acid methyl esters (FAMEs), the lipid extract was acid-catalyzed esterified in 4:1 (*v*/*v*) methanol-hexane with acetyl chloride at 100 °C for 1 h, followed by the addition of potassium carbonate for 5 min at ambient temperature to neutralize the reaction. Then, the FAME in the hexane phase was collected and further analyzed through GC-FID analysis using GC-2010 Plus (Shimadzu, Kyoto, Japan), equipped with a DB-23 capillary column (Agilent Technologies, Santa Clara, CA, USA) with an injection temperature at 250 °C. For the column temperature program, the initial temperature was set at 180 °C, held for 15 min, and increased to 220 °C at a heating rate of 4 °C/min, and finally held for 7 min at 220 °C. Helium was used as the carrier gas at a flow rate of 62.9 mL/min [37]. The fatty acid profile of *A. mellifera* brood fat extracts was analyzed in comparison with that of commercial beeswax. The analysis was performed using three independent sample replicates.

### 2.5. Characterization of A. mellifera Brood Fat Extracts

#### 2.5.1. Functional Groups Determination by Fourier Transform Infrared Spectrophotometer (FTIR)

Functional groups of the *A. mellifera* brood fat extracts were identified by FTIR. The aliquot amount (1 g) of each fat extract was subjected to the FTIR spectrometer (Alpha-II, Bruker, Karlsruhe, Germany), equipped with the single reflection diamond attenuated total reflection (ATR) module, and the FTIR spectra of all samples were subsequently recorded at 4 mm/s across a range of wavenumbers, scanning from 400 to 4000 cm^−1^. The FTIR spectra were plotted, with transmittance represented on the *Y*-axis and the wavenumber (cm^−1^) plotted along the *X*-axis. The analysis was performed using three independent sample replicates.

#### 2.5.2. Crystalline Structure Study by X-Ray Diffractometer (XRD)

Crystallographic analysis of the *A. mellifera* brood fat extracts was carried out by XRD (D2 PHASER, Bruker, Karlsruhe, Germany), equipped with a copper radiation source (λ = 1.54 Å). Firstly, each extract was put into a 25 mm specimen ring which was further placed on the XRD holder and sample stage within the XRD chamber, respectively. The scanning was performed at a 2θ range from 5 to 80°, with a time per step of 0.2°·s^−1^ [38]. The resulting XRD spectra were plotted with intensity (arbitrary units, a.u.) values on the *Y*-axis and the 2θ angle (degrees) on the *X*-axis. The analysis was performed using three independent sample replicates.

#### 2.5.3. Thermal Behavior Analysis by Differential Scanning Calorimeter (DSC) and Thermogravimetric Analyzer (TGA)

Thermal analysis was investigated in order to evaluate lipid crystallinity, polymorphism, and thermal properties of each *A. mellifera* brood fat extracts by using DSC (DSC25, TA Instruments, New Castle, DE, USA) and TGA (TGA550, TA Instruments, New Castle, DE, USA). Briefly, 8–9 mg of each sample was accurately weighed into standard pans, and the device was set within the range of 25 to 550 °C for DSC and 50 to 800 °C for TGA measurement with a scan rate of 10 °C/min. An empty standard pan was used as a reference [12,30]. All measurements were carried out under a nitrogen atmosphere using three independent sample replicates.

### 2.6. Irritation Test by Hen’s Egg Test Chorioallantoic Membrane (HET-CAM) Assay

*A. mellifera* brood fat extracts were tested for their irritation potential by the HET-CAM assay following a procedure earlier described by Chaiyana et al. [39]. Briefly, The CAM of fertilized hen eggs aged between 7 and 9 days was prepared by cutting the eggshell to indicate the aerobic part of the inner membrane, which was exposed to 0.9% (*w*/*v*) NaCl to maintain appropriate moisture. The eggs were then placed in the hatching machine (Nanchang Howard Technology Co., Ltd., Jianxi, China) at a temperature of 37.5 ± 0.5 °C for 15 min. Following this, the inner layer of the eggshell was carefully removed, and 30 µL of each sample was dropped onto the CAM. The irritation effects were immediately observed for 5 min and 60 min, respectively. Irritation properties were described as irritation score (IS) and calculated using the following equation:

IS = [(301 − H)/300 × 5] + [(301 − L)/300 × 7] + [(301 − C)/300 × 9](2)

where H denotes the beginning of vascular hemorrhage, L denotes the beginning of vascular lysis, and C denotes the beginning of vascular coagulation on the CAM. The IS was classified as follows: 0 = (0.0–0.9) as non-irritating, 1 = (1.0–4.9) as slightly irritative, 2 = (5.0–8.9) as moderately irritative, and 3 = (9–21) as strongly irritative. A solution of 1% (*w*/*v*) SLS was used as a positive control and a normal saline solution (NSS) was used as a negative control. IPM was used as a vehicle control and commercial beeswax was evaluated as a reference natural wax. Three experiments were performed independently.

### 2.7. Determination Anti-Inflammatory Activities of A. mellifera Brood Fat Extracts

The anti-inflammatory activity of *A. mellifera* brood fat extracts was determined in terms of IL-6 and TNF-α inhibition using the ELISA technique according to the manufacturer’s product information sheet (Invitrogen, Grand Island, NY, USA).

#### 2.7.1. Murine Macrophage Cell Line (RAW 264.7) Culture

The RAW 264.7 murine macrophage cell line (Accession No. TIB-71), obtained from the American Type Culture Collection (ATCC, Manassas, VA, USA), was cultured in 10% FBS in DMEM and supplemented with 100 U/mL penicillin and 100 µg/mL streptomycin. The cells were maintained in a CO_2_ incubator (SHEL LAB Model 3503, Sheldon Manufacturing, Inc., Orlando, FL, USA) at 37 °C in a 5% CO_2_ moist atmosphere (90% relative humidity).

#### 2.7.2. Determination of Cytotoxicity

The cytotoxicity of *A. mellifera* brood fat extracts on RAW 264.7 cells was evaluated using the MTT assay. Firstly, the cells at a density of 5 × 10^4^ cells/cm^3^ were seeded in 96-well plates and incubated in 10% FBS in DMEM at 37 °C, 5% CO_2_, and 90% relative humidity for 24 h. On the second day, 100 µL of each sample at final concentrations of 3.125, 6.25, 12.5, 25, 50, and 100 µg/mL in 10% FBS in DMEM were added to the wells, with EA included as the vehicle control and without it for the cell control. The treated cells were further incubated under the same conditions for 48 h. Then, 15 µL of 5 mg/mL of MTT dye solution was added into each well followed by further incubation for 4 h. After removal of the supernatant, the formazan crystals formed at the bottom of the wells were dissolved by adding DMSO. Finally, the optical density was measured using an ELISA plate reader (SPECTROstar^®^ Nano, BMG Labtech GmbH, Ortenberg, Germany) at 578 nm with a reference wavelength number at 630 nm. Three independent experiments were performed. The percentage of cell viability was calculated using the following equation:

Cell viability (%) = A/B × 100(3)

where A indicates the average absorbance in the cells treated with samples and B is the average absorbance in the vehicle control well. The analysis was performed using three independent sample replicates, with each sample analyzed in triplicate.

#### 2.7.3. Determination of IL-6 and TNF-α Inhibition by ELISA

The anti-inflammatory activities of *A. mellifera* brood fat extracts were evaluated based on IL-6 and TNF-α inhibition using an ELISA technique according to the manufacturer’s product information sheet (Invitrogen, Grand Island, NY, USA). Briefly, the RAW 264.7 cells at a density of 5 × 10^4^ cells/well were seeded in 24-well plates and incubated in 10% FBS in DMEM at 37 °C, 5% CO_2_, and 90% humidity for 24 h. The following day, *A. mellifera* brood fat extracts were applied to RAW 264.7 cells and further incubated at the same conditions for 2 h. Then, 1 µg/mL of LPS was added and further incubated at the same conditions for 24 h. On the third day, the supernatant was collected and further analyzed for the secretion of IL-6 and TNF-α by the ELISA [40]. All incubation steps were performed at room temperature. The optical density at 450 nm, corrected by the reference wavelength 630 nm, was measured with the ELISA microplate reader (SPECTROstar^®^ Nano, BMG Labtech GmbH, Ortenberg, Germany). Cells treated with dexamethasone, commonly known as a potent steroid used in anti-inflammation, served as a positive control, while cells treated with LPS served as a control. The percentage of cell viability was calculated using the following equation:

IL-6 or TNF- α inhibition (%) = [(A − B)/A) × 100](4)

where A indicates the average absorbance of the control wells and B represents the average absorbance of cells treated with the samples. The commercial beeswax was also evaluated as a reference natural wax. All experiments were performed using three independent sample replicates, with each sample analyzed in triplicate.

### 2.8. Development of NLC from A. mellifera Brood Fat Extracts

Prior to NLC development, the required hydrophilic–lipophilic balance (rHLB) of each *A. mellifera* brood fat extract was evaluated following the method of Chaiyana et al. (2024), and the results indicated that all extracts exhibited an equivalent rHLB value of 11 [41]. In brief, surfactant mixtures of Tween^®^ 80 (HLB 15) and Span^®^ 80 (HLB 4.3) covering an HLB range of 4.3–15.0 were prepared at a total concentration of 5% *w*/*w* and used to formulate oil–water emulsions. The emulsions were vortex-mixed for 10 min and evaluated for phase separation immediately after preparation and after storage at room temperature for up to 24 h, with phase separation at 24 h used to calculate the rHLB value [41]. Therefore, a combination of Tween^®^ 80 and Span^®^ 80, providing an HLB value of 11, was used as the surfactant system at a concentration of 5% *w*/*w* for NLC development. *A. mellifera* brood fat extracts were used as the solid lipid in comparison with commercial beeswax, while sugar squalane served as the liquid lipid. The effects of solid lipid-to-liquid lipid ratios of 5:0, 3.5:1.5, 2.5:2.5, and 1.5:3.5 *w*/*w* on NLC formation were investigated. NLCs from *A. mellifera* brood fat extracts were developed using the probe sonication method [42]. In brief, the primary emulsion was prepared as follows. First, an aqueous phase containing Tween^®^ 80 and DI water was mixed and heated to 85 °C. The lipid phase, consisting of *A. mellifera* brood fat extracts, sugar squalane, and Span^®^ 80, was mixed and heated to 80 °C. The heated aqueous phase was then added to the lipid phase, and the resulting mixture was immediately subjected to a probe sonicator (Q125, Qsonica, Newton, CT, USA) using a 10 s pulse-on and 2 s pulse-off mode at 80 W for 5 min. Additionally, the high-pressure homogenization method was evaluated. This involved processing the primary emulsion through a high-pressure homogenizer (APV 1000, Wilmington, MA, USA) at 200 bars for 7 cycles [30]. NLCs processed by probe sonication were prepared in 30 g batches, whereas those processed by high-pressure homogenization (HPH) were prepared in 300 g batches.

### 2.9. Characteriztion and Stability of NLC from A. mellifera Brood Fat Extracts

#### 2.9.1. Determination of Particle Size, Zeta Potential, and Polydispersity Index (PDI)

All NLCs were characterized for their particle size, PDI, and zeta potential using dynamic light scattering (DLS) by a photon correlation spectroscopy (Zetasizer ZS, Malvern Instruments Ltd., Malvern, UK). Before the measurements, the formulations were diluted 100 times with DI water. Sizing and polydispersity index measurements were carried out at a fixed angle of 173°, while zeta potential was determined by electrophoretic light scattering, using a Zetasizer ZS (Malvern Instruments Ltd., Malvern, UK). All experiments were performed in triplicate.

#### 2.9.2. Stability Test

The NLC formulations were subjected to an accelerated stability test involving eight heating-cooling cycles, with each cycle consisting of 24 h at 4 °C and 24 h at 45 °C. The formulations were also monitored for long-term stability by storage for one month at three distinct storage conditions, 4 °C, 45 °C, and room temperature under ambient humidity. Subsequently, each NLC formulation was characterized for their particle size, PDI, and zeta potential using the techniques mentioned above in Section 2.9.1.

#### 2.9.3. Morphology Determination

The NLC with favorable characteristics (small particle size, narrow PDI, and pronounced zeta potential) and stability were evaluated for their morphology using transmission electron microscope (TEM, JEM-2100 Plus, JEOL, Tokyo, Japan). Briefly, after the NLCs dispersion has been diluted with DI water at a 1:10 (*v*/*v*) ratio [43], a single drop of NLC was put onto a copper grid and allowed to air-dry at room temperature. Subsequently, the grid was negatively stained with 1% phosphotungstic acid. Prior to analysis, the copper grid was then be allowed to dry at room temperature. The particle size of the NLCs was analyzed from TEM micrographs using ImageJ (version 1.54g, Wayne Rasband and contributors, National Institutes of Health, Bethesda, MD, USA). The image scale was calibrated based on the scale bar provided in the TEM images using the Set Scale function. The images were converted to 8-bit grayscale, and threshold adjustment was performed to enhance the contrast between nanoparticles and the background. Individual particle diameters were measured by drawing a straight line across the widest part of each nanoparticle. The measured diameters were recorded, and the particle size distribution was presented as a histogram.

#### 2.9.4. Irritation Test by HET-CAM Assay

The NLC with favorable characteristics and stability were evaluated for their irritation potential using HET-CAM assay [39], as described in Section 2.6.

#### 2.9.5. Determination of Anti-Inflammatory Activity

The NLCs with favorable characteristics and stability were evaluated for their anti-inflammatory activities by measuring the inhibition of IL-6 and TNF-α using an ELISA [40], as described in Section 2.7.

### 2.10. Statistical Analysis

All data are shown as mean ± SD. Differences between two groups were analyzed using an unpaired Student’s *t*-test, while comparisons among multiple groups were analyzed using one-way analysis of variance (ANOVA), followed by Tukey’s post hoc test, conducted using GraphPad Prism software version 2.01 (GraphPad Software Inc., La Jolla, CA, USA). The level of significance was set at *p* < 0.05.

## 3. Results and Discussions

### 3.1. A. mellifera Brood Fat Extracts

Fat extracts from *A. mellifera* brood were successfully obtained using AC, EA, and HX extraction, and all extracts appeared as yellow to yellow-orange semisolid materials at ambient temperature (Table 1). The semisolid consistency observed in all samples suggests a high lipid content, as fats rich in long-chain fatty acids and waxy components typically exhibit solid or semisolid characteristics at ambient temperature, consistent with previous reports identifying lipids as major constituents of *A. mellifera* brood [44]. However, the AC extract exhibited a brownish-yellow color, whereas the EA and HX extracts were lightly yellow. The difference may be attributed to the use of AC, a semipolar solvent capable of extracting polar compounds, including pigments, which likely contributed to the darker coloration of the AC extract compared with the others [45,46]. The extraction yields varied significantly depending on the solvent used as shown in Table 1. The highest yield was obtained in EA (29.0 ± 1.0% *w*/*w*), followed by HX (27.8 ± 0.4% *w*/*w*), while AC yielded the lowest extraction efficiency (22.8 ± 0.0% *w*/*w*). The significantly higher yields obtained with EA and HX may be attributed to their greater affinity for nonpolar lipid components present in the brood matrix, including triglycerides, wax esters, and certain phospholipids, which are abundantly found in the larval stage of holometabolous insects [47]. As AC is moderately polar, it may be less effective in solubilizing highly hydrophobic lipid fractions, resulting in a lower extraction yield. Interestingly, EA provided a yield comparable to that of HX while offering a relatively greener and less toxic solvent alternative [48,49]. Consistent with previous studies, EA has been reported as a promising alternative to HX for lipid extraction from oilseeds, offering comparable or higher oil yields and similar quality parameters, while providing improved safety and reduced environmental impact [48]. Additionally, EA has been identified as the best alternative solvent to HX for the extraction of salmon oil lipids [49]. The current study also suggested that EA may be a suitable solvent for extracting brood-derived lipids from insect materials, in addition to plant or fishery sources.

### 3.2. Fatty Acid Profile of A. mellifera Brood Fat Extracts

The fatty acid composition of each *A. mellifera* brood fat extract was compared with beeswax, a common wax used in cosmetic and pharmaceutical applications [50]. The findings are shown in Table 2 highlighting the differences in fatty acid composition between *A. mellifera* brood fat extracts and commercial beeswax. Beeswax was characterized by a significantly higher proportion of medium- and long-chain saturated fatty acids (SFA), especially palmitic acid (47.9% *w*/*w*) and lignoceric acid (19.8% *w*/*w*), resulting in a total SFA content of 73.6% *w*/*w*. The high level of these saturated fatty acids is consistent with the well-known rigid and waxy nature of beeswax and its structural role in the hive [51,52]. The findings were in agreement with the study of Jimenez et al. (2006), which identified palmitic acid and lignoceric acid as the major fatty acids in beeswax [53]. Similarly, Navarro-Hortal et al. (2019) identified hydrocarbons and monoesters as the main components of beeswax, which are predominantly saturated [54].

In contrast, all *A. mellifera* brood fat extracts contained significantly lower SFA contents (57.9–59.3% *w*/*w*) and markedly higher proportions of unsaturated fatty acids (USFA) (40.7–42.1% *w*/*w*) compared with beeswax. Among the SFA, palmitic acid was the predominant component in all *A. mellifera* brood fat extracts (42.6–43.9% *w*/*w*), while other SFAs were present only in minor amounts, and stearic acid levels (9.8–10.1% *w*/*w*) were significantly higher than those observed in beeswax. Notably, oleic acid was the dominant fatty acid in brood fat extracts (38.9–40.3% *w*/*w*), whereas its concentration in beeswax was substantially lower (21.4% *w*/*w*). The differences in fatty acid profiles were consistent with the distinct external appearances of beeswax and *A. mellifera* brood fat extracts. Beeswax, which contained a high proportion of medium- and long-chain SFA, exhibited a solid and rigid structure at ambient temperature [55]. In contrast, *A. mellifera* brood fat extracts are characterized by higher levels of USFA, particularly oleic acid, together with shorter-chain SFA such as palmitic and stearic acids, resulting in a semisolid lipid matrix [56].

The different extraction solvents were also found to affect the fatty acid composition of *A. mellifera* brood fat. Although palmitic acid and oleic acid were the primary fatty acids in all extracts in the current study, consistent with previous reports [57,58], variations were observed depending on the solvent used. EA and HX, both considered low-polarity solvents, efficiently extracted medium- to long-chain saturated lipids [49,59]. In contrast, AC yielded a slightly different lipid profile, producing the highest amount of oleic acid, in agreement with previous findings that its high polarity favors the solubilization of USFA and more oxygenated lipid species [60]. It should be noted that EA and HX extract were comparable in fatty acid profiles, which differed slightly from that of the AC extract. Therefore, EA could serve as a greener alternative to HX, efficiently extracting medium- to long-chain saturated lipids while offering a more environmentally friendly option [48,49]. Both solvents yielded similar extraction efficiencies and fatty acid profiles.

### 3.3. Physicochemical Properties of A. mellifera Brood Fat Extracts

**Figure 2:**
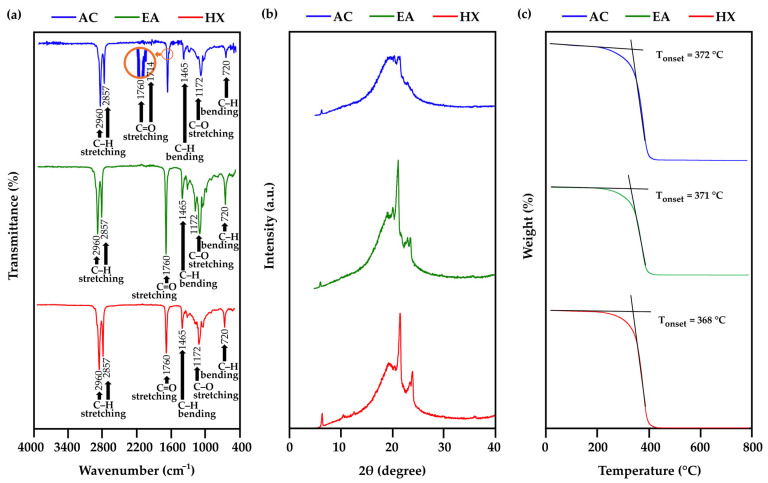
FTIR spectra (**a**), XRD spectra (**b**), and TGA thermogram (**c**) of *A. mellifera* brood fat extracts extracted using acetone (AC), ethyl acetate (EA), and hexane (HX). The highlighted region in the orange circle represents a magnified view of selected absorption bands. The black arrows indicate characteristic peaks corresponding to specific functional groups.

*A. mellifera* brood fat extracts were evaluated for their physicochemical properties in comparison with beeswax, with a focus on molecular composition (functional groups) assessed by FTIR, crystalline characteristics examined by XRD, and thermal stability determined by TGA, as shown in Figure 2.

The FTIR spectra of *A. mellifera* brood fat extracts obtained using different solvents, as shown in Figure 2a, exhibited characteristic absorption bands corresponding to the major functional groups of lipids. A broad absorption in the range ~2960–2857 cm^−1^, observed for all extracts, corresponded to the asymmetric and symmetric stretching vibrations of CH_3_ and CH_2_ groups in the hydrocarbon chains of fatty acids, which form the backbone of triacylglycerols commonly found in natural lipids and oils [61,62]. The strong band near 1760 cm^−1^ corresponds to the C=O stretching of ester linkages between fatty acids and the glycerol backbone, a key feature of both animal and plant waxes [12,63]. The presence of peaks at 1714 cm^−1^ indicates C=O stretching of free fatty acids [64]. However, this band appears less distinct because it is partially overlapped by the strong ester C=O stretching band around 1740 cm^−1^. A slight indication of this band can be observed in the AC extract, whereas in the other extracts it is only visible as a weak shoulder. This difference may be attributed to the higher polarity of AC (dielectric constant of 20.7 at 25 °C [65,66]) compared with EA (dielectric constant of 6.02 at 25 °C [66]) and HX (dielectric constant of approximately 1.89 at 25 °C [67]), which favors the extraction of relatively more free fatty acids in the AC extract. In contrast, the less polar solvents (EA and HX) preferentially extract non-polar lipid components, such as wax esters, resulting in a dominant ester carbonyl band around 1740 cm^−1^ that partially masks the weaker free fatty acid signal at 1714 cm^−1^. Bending vibrations of CH_2_ and CH_3_ groups were observed at 1465 cm^−1^ (scissoring) and 720 cm^−1^ (rocking), consistent with long-chain saturated fatty acids [64]. C–O stretching vibrations of ester groups appeared at 1172 cm^−1^ [68], while the fingerprint region (1500–1000 cm^−1^) displayed additional characteristic skeletal vibrations of methylene groups. The consistent FTIR patterns of all *A. mellifera* brood fat extracts reflect conserved chemical profiles of the extracts, with similar triacylglycerol backbones and ester linkages, supported by fatty acid profiles dominated by palmitic, stearic, and oleic acids corresponding to the characteristic aliphatic C–H and carbonyl bands.

Interestingly, the FTIR spectral profiles of the *A. mellifera* brood fat extracts and beeswax [64], as summarized in Table 3, are highly similar with nearly identical characteristic absorption bands. Although the fatty acid profiles of the *A. mellifera* brood fat extracts differed distinctly from those of beeswax, the FTIR spectra of all extracts remained remarkably similar. This apparent contradiction can be explained by the fact that FTIR primarily detected functional groups and overall molecular structures rather than variations in individual fatty acid composition [69]. The characteristic absorption bands for CH_3_/CH_2_ stretching, ester C=O stretching, and C–O vibrations are common to most long-chain fatty acids and triacylglycerols [11], which dominate the extracts. Therefore, even if the relative proportions of specific fatty acids (e.g., palmitic, stearic, oleic acids) differ between samples, the overall chemical backbone remains similar, resulting in near-identical FTIR profiles. This emphasized that while FTIR was excellent for identifying general lipid structures and functional groups, it might not capture fine compositional differences, which were better revealed through detailed fatty acid profiling techniques such as GC–MS. However, FTIR has been proposed as a rapid and reliable method for assessing beeswax authenticity and detecting adulteration, such as paraffin addition, by monitoring characteristic functional group ratios (e.g., C=O stretching vs. CH_2_/CH_3_ vibrations), which are sensitive to even small amounts of adulterant and largely unaffected by routine heating/cooling [69].

The crystalline characteristics of the *A. mellifera* brood fat extracts were also evaluated. The XRD diffractograms of each extract, as shown in Figure 2b, revealed that all samples exhibited a dominant broad diffraction halo centered around 2θ ≈ 18–22°, which is characteristic of predominantly amorphous structures. Additionally, strong crystalline peaks at diffraction angles of 21.5° and 23.9° were observed in the XRD diffractograms of the HX and EA extract, but were absent in AC extract, indicating that the AC extract has a markedly lower crystallinity compared with the HX and EA extract. This difference can be attributed to the polarity-dependent extraction behavior of the solvents. HX and EA, owing to their relatively low polarity, preferentially extract nonpolar constituents such as medium- and long-chain fatty acids, which are known to form ordered crystalline structures and exhibit characteristic diffraction peaks in the range of 2θ ≈ 21–23° [70,71]. In contrast, the higher polarity of AC favors the extraction of more polar components, which may result in a predominantly amorphous structure. Previous studies have shown that polar lipids disrupt crystallization kinetics by acting as molecular spacers that interfere with triacylglycerol alignment, reduce nucleation efficiency, and delay polymorphic transitions toward more stable crystalline phases [72]. These findings demonstrate that solvent selection significantly influences the solid-state characteristics of the extracts, which may subsequently impact their physicochemical properties and functional performance. In comparison with the *A. mellifera* brood fat extracts obtained using different solvents, beeswax exhibited a markedly higher degree of structural organization, reflecting its inherently semicrystalline nature and well-ordered molecular packing [73]. Beeswax is a semicrystalline solid, the structural organization of which was evidenced by strong diffraction reflections at 2θ ≈ 21.5° and 24°, together with weaker reflections at approximately 30° and 36.2°, which were characteristic of an orthorhombic arrangement [73]. The presence of these well-defined diffraction peaks confirmed a high degree of molecular ordering in beeswax, which accounted for its mechanical rigidity and solid state at room temperature, in contrast to the *A. mellifera* brood fat extracts, which exhibited lower crystallinity and a semisolid nature.

The thermal stability of *A. mellifera* brood fat extracts obtained using different solvents was evaluated by TGA, as shown in the thermograms in Figure 2c. All extracts exhibited a similar thermal degradation profile, with no significant mass loss observed at lower temperature ranges, while the onset of decomposition occurring at approximately 370 °C, followed by a rapid mass loss up to 400 °C. This behavior is consistent with the corresponding DSC thermograms (Figure S1), which displayed a broad endothermic event in the temperature range of 390–420 °C, indicating similar thermal transitions associated with decomposition processes. The observed thermal degradation is primarily attributed to the decarboxylation of fatty acid chains during thermal decomposition [74,75]. These results suggest that the extraction solvent had only a very small influence on the thermal stability of the brood fat extracts, as all samples exhibited comparable decomposition temperature ranges. Additionally, the absence of weight loss in the lower temperature region suggested that no detectable non-lipid residual impurities were present in the extracts. However, the thermal stability of *A. mellifera* brood fat extracts differed from that of beeswax reported in the literature, which typically exhibited two major weight-loss stages at approximately 360–380 °C and 450–480 °C and thermal stability up to about 550 °C under an inert atmosphere [76]. This distinction highlights again the differences in lipid compositions between the *A. mellifera* brood fat extracts and beeswax, which influenced their thermal degradation behavior.

### 3.4. Irritation Properties of A. mellifera Brood Fat Extracts

**Figure 3:**
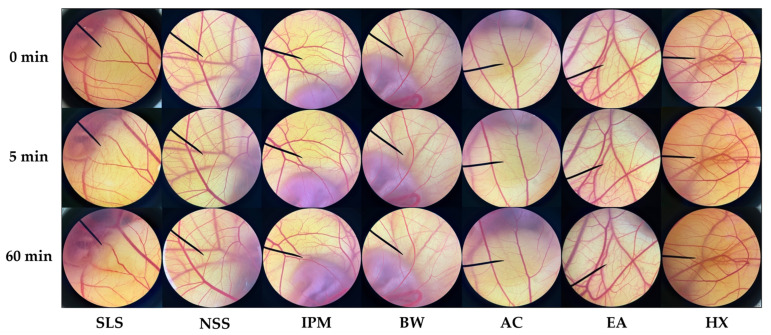
Chorioallantoic membrane after exposure to different treatments, including positive control (1% *w*/*v* sodium lauryl sulfate, SLS), negative control (normal saline solution, NSS), vehicle control (isopropyl myristate, IPM), commercial beeswax (BW), and *A. mellifera* brood fat extracts—acetone extract (AC), ethyl acetate extract (EA), and hexane extract (HX). The letter H represents vascular hemorrhage, C represents vascular coagulation, and L represents vascular lysis.

*A. mellifera* brood fat extracts were evaluated for their irritation potential in comparison with beeswax, as shown in Figure 3, with the respective irritation scores listed in Table 4. Since beeswax and *A. mellifera* brood fat extracts were solid and semisolid at room temperature, they were poorly soluble in water and require a suitable lipophilic vehicle to form a uniform dispersion. IPM was chosen because it is a non-polar and skin-compatible ester that can efficiently dissolve solid and semisolid lipids, enabling homogeneous incorporation into formulations. Additionally, IPM is widely used in topical and cosmetic products due to its low viscosity, good spreading properties, and non-greasy feel [77]. The findings demonstrated that all *A. mellifera* brood fat extracts, as well as commercial beeswax and IPM, exhibited excellent safety, producing irritation scores of 0.0 ± 0.0, comparable to the negative control, NSS, which is isotonic. No signs of hemorrhage, coagulation, or vessel lysis were observed, indicating that these fat extracts were inherently non-irritating to the chorioallantoic membrane. In contrast, the positive control (1% *w*/*v* SLS) induced pronounced irritation responses, including hemorrhage, coagulation, and vessel lysis, within 5 min of exposure, with severity increasing over time (IS = 17.7 ± 1.0). The HET-CAM assay, originally used for eye irritation testing [78], is widely adopted for evaluating skin irritation due to its high sensitivity, rapid results, and low cost [79]. Using embryos that have not reached half of the gestation period, it can be conducted without ethical approval, making it a practical and reliable tool for preliminary screening of topical formulations, such as *A. mellifera* brood fat extracts [80]. Therefore, the findings confirm that *A. mellifera* brood fat extracts are safe and well-tolerated for topical use, supporting their potential as biocompatible lipid excipients and their suitability for further development in topical formulations.

### 3.5. Anti-Inflammatory Properties of A. mellifera Brood Fat Extracts

**Figure 4:**
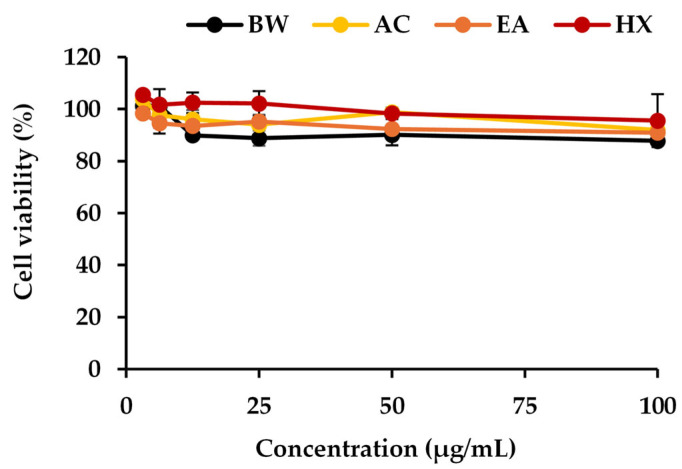
Cytotoxic effects of commercial beeswax (BW) and *A. mellifera* brood fat extracts extracted using acetone (AC), ethyl acetate (EA), and hexane (HX) on RAW 264.7 cells.

The cytotoxicity of *A. mellifera* brood fat extracts on RAW 264.7 cells was evaluated over a concentration range of 3.125–100 µg/mL, as shown in Figure 4. RAW 264.7 macrophages were used as a representative immune cell model due to their central role in the body’s first line of defense against foreign materials [81]. These cells are highly responsive to inflammatory stimuli, producing reactive oxygen and nitrogen species as well as key pro-inflammatory cytokines such as IL-6 and TNF-α, which are critical markers for assessing anti-inflammatory activity [82]. Cell viability remained above 80% after 48 h of exposure to all *A. mellifera* brood fat extracts, indicating that the extracts did not exert significant cytotoxic effects at the tested concentrations. These findings demonstrate that *A. mellifera* brood fat extracts are well-tolerated by RAW 264.7 macrophages, supporting their suitability for subsequent evaluation of anti-inflammatory activity. The absence of cytotoxicity is particularly important for topical and dermal applications, where preserving cell viability is essential to maintain normal skin immune responses. Moreover, these results suggest that the extracts can be safely incorporated into topical formulations without inducing adverse cellular effects.

**Figure 5:**
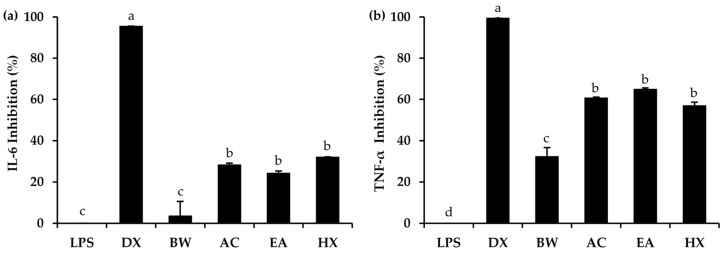
Inhibitory activities on IL-6 (**a**) and TNF-α (**b**) in RAW 264.7 cells stimulated with lipopolysaccharide (LPS) following treatment with dexamethasone (DX), commercial beeswax (BW), and *A. mellifera* brood fat extracts extracted by acetone (AC), ethyl acetate (EA), and hexane (HX). Superscript letters a, b, c, and d indicate significant differences in inhibitory activities among treatments, as determined by one-way ANOVA with Tukey’s post hoc test (*p* < 0.05).

The anti-inflammatory potential of *A. mellifera* brood fat extracts was evaluated in RAW 264.7 macrophages using LPS, a well-known pro-inflammatory activator, to induce macrophage-mediated inflammatory responses [81]. IL-6 and TNF-α, key pro-inflammatory cytokines secreted by macrophages, were measured to evaluate the anti-inflammatory effects of *A. mellifera* brood fat extracts, as shown in Figure 5. The results demonstrated that dexamethasone, a known anti-inflammatory agent serving as a positive control, exhibited the highest inhibition of both IL-6 (95.5 ± 0.0%) and TNF-α (99.4 ± 3.6%), confirming the responsiveness of the system. All *A. mellifera* brood fat extracts significantly suppressed cytokine production and were more potent than beeswax (*p* < 0.05). All three extracts reduced IL-6 (AC extract: 28.2 ± 0.9%, EA extract: 24.1 ± 1.2%, HX extract: 32.0 ± 0.2%) and TNF-α (AC extract: 60.7 ± 0.4%, EA extract: 64.9 ± 0.7%, HX extract: 56.9 ± 1.8%), whereas beeswax showed minimal IL-6 inhibition (3.5 ± 3.1%) and moderate TNF-α suppression (32.3 ± 4.3%). These findings indicate that *A. mellifera* brood fat extracts had greater anti-inflammatory potential than beeswax and may serve as promising natural anti-inflammatory agents in pharmaceutical and cosmetic applications. The observed anti-inflammatory activity of *A. mellifera* brood fat extracts can be attributed primarily to their high oleic acid content, which has been widely reported to modulate multiple inflammatory pathways [83,84,85,86]. Oleic acid has been reported to suppress the production of pro-inflammatory mediators including IL-6, TNF-α, iNOS, and COX2 in LPS-stimulated monocytes and macrophages [82,83,84,85,86]. Mechanistically, oleic acid inhibited Toll-like receptor (TLR3 and TLR4) signaling, preventing the downstream activation of nuclear factor kappa B (NF-κB) and mitogen-activated protein kinase (MAPK) pathways, which are key regulators of cytokine expression [87]. Additionally, oleic acid can activate peroxisome proliferator-activated receptors (PPARs), enhancing the expression of antioxidant enzymes such as catalase and superoxide dismutase, thereby reducing intracellular reactive oxygen species and further suppressing NF-κB-mediated cytokine release [88,89]. Some studies also suggested that oleic acid may directly interact with NF-κB to promote its proteolytic degradation, contributing to the inhibition of pro-inflammatory signaling [90,91].

### 3.6. NLC from A. mellifera Brood Fat Extracts

For NLC development, *A. mellifera* brood fat extract was employed as the solid lipid and sugar squalane as the liquid lipid. Sugar squalane was selected due to its sebum-mimetic nature and its ability to preserve skin barrier homeostasis, making it particularly advantageous for photoaging prevention, sensitive skin, and long-term dermal applications [92]. In addition, sugar squalane is derived from renewable sugar-based feedstocks, aligning with the sustainability concept. This integrated approach reinforces the overall green and sustainable formulation strategy adopted in the present study.

Prior to NLC development, the rHLB values of the *A. mellifera* brood fat extracts were determined and were found identical across all samples, with all extracts exhibiting an rHLB value of 11. The surfactant system (Tween^®^ 80 and Span^®^ 80) with an HLB value of 11 successfully emulsified the NLCs, producing stable dispersions across all lipid types and solid-to-liquid ratios. The particle size, PDI, and zeta potential of these NLCs, along with those of NLCs prepared from commercial beeswax at the same ratios, are summarized in Table 5.

The solid-to-liquid lipid ratio significantly influenced NLC characteristics. The formulation without sugar squalane as the liquid lipid, classified as a solid lipid nanoparticle (SLN) representing the first generation of lipid nanoparticles composed solely of solid lipid [93], exhibited the largest particle sizes compared with the other NLC formulations (*p* < 0.05). Among the SLNs, the largest particle size and the widest PDI were observed for the AC extract, which may be related to differences in lipid crystallinity compared with the EA and HX extracts. Although the fatty acid profiles of the different *A. mellifera* brood fat extracts were generally comparable, slight variations in their composition were observed. This observation is consistent with previous studies reporting that SLNs formulated with different fatty acids can exhibit variations in particle size and PDI due to differences in carbon chain length and physicochemical properties [94]. In addition, variations in crystallinity were observed, with the AC extract exhibiting a more amorphous structure. This may be attributed to the ability of AC to extract a broader range of polar and semi-polar lipid components, which can interfere with lipid crystallization and nanoparticle formation during the preparation of SLNs.

Increasing the proportion of liquid lipid consistently reduced particle size and PDI for all lipid types, indicating that a higher liquid lipid content enhances lipid fluidity, improves emulsification, and reduces particle aggregation [95]. For example, beeswax-based NLCs decreased in size from 355.1 ± 2.0 nm at a 5:0 ratio to 93.7 ± 0.6 nm at a 1.5:3.5 ratio, and a similar trend was observed for all *A. mellifera* brood fat extracts. In addition, PDI values also decreased with increasing liquid lipid content, reflecting more homogeneous dispersions. However, the zeta potential showed less consistent trends with lipid ratio but although some differences were observed among NLC formulations, the overall range was relatively narrow (−25.2 ± 0.6 to −36.7 ± 1.0), suggesting only minor variations on electrostatic stability.

Among the different fat types, *A. mellifera* brood fat extracts produced NLCs with smaller particle sizes, lower PDI, and more negative zeta potentials compared with commercial beeswax at the same solid-to-liquid lipid ratios. The PDI of *A. mellifera* brood fat extract NLCs was also lower, indicating more homogeneous dispersions, whereas beeswax NLCs exhibited higher PDI values, suggesting a broader particle size distribution. As stated above, with respect to the zeta potential, the type of fat had little effect. However, these findings indicated that lipid type was a critical factor affecting NLC formation, particle uniformity and stability. All *A. mellifera* brood fat extract-based NLCs demonstrated more favorable physicochemical characteristics, including smaller particle size, narrower PDI, and suitable zeta potential in every solid-to-liquid lipid ratio when compared with beeswax-based NLCs. These findings could be attributed to the high oleic acid content in *A. mellifera* brood fat extracts, which reduces NLC core viscosity and promotes a less structured crystal lattice, thereby enhancing lipid fluidity and enabling the formation of smaller particles [96,97,98].

Among the lipid types investigated, EA extracts emerged as the best choice for NLC preparation. EA extract-based NLCs consistently produced smaller particle sizes compared with AC and HX extracts, and commercial beeswax at the same solid-to-liquid lipid ratios, which would be advantageous for stability, skin penetration, and bioactive delivery [99]. These NLCs also exhibited low PDI values, indicating homogeneous particle distributions, and sufficiently negative zeta potentials, supporting electrostatic stabilization. In addition, considerations of extraction yield and biological activity further support the selection of the EA extract, making it the most suitable lipid for subsequent NLC development and optimization. Additionally, the 3.5:1.5 solid-to-liquid lipid ratio was selected for further NLC evaluation due to its higher solid lipid content, which is likely to enhance the structural integrity and encapsulation efficiency of the nanoparticles [100]. Previous studies have reported that higher solid lipid content enhanced entrapment efficiency by forming compact, densely packed, and orderly lipid matrices that more effectively retain drug molecules compared with less ordered systems [100]. A more rigid lipid matrix at higher lipid ratio could better retain and protect bioactive compounds, while also enabling controlled or sustained release [101]. Although the particle size at a solid-to-liquid lipid ratio of 3.5:1.5 is slightly larger than that observed at 2.5:2.5, the NLCs still exhibit reasonably small sizes and low PDI values, ensuring good homogeneity and stability.

Aside from the effects of NLC composition, the influence of the particle size reduction method on the physicochemical properties of the formulations was also evaluated. To this end, probe sonication and high-pressure homogenization were compared, and the results are summarized in Table 6. The formulation prepared by high-pressure homogenization exhibited a smaller particle size (72.1 ± 0.3 nm) compared with the formulation prepared using the probe sonication method (108.0 ± 0.6). The superior performance of high-pressure homogenization is attributed to the generation of strong shear forces, turbulence, and cavitation, which reduce particle size to the nanometer range by forcing the pre-emulsion of lipids and aqueous phase through a narrow gap at high pressure [102]. Both formulations, however, exhibited very low PDI values around 0.1, indicating narrow particle size distributions, high uniformity, and good colloidal stability, thereby minimizing the tendency toward particle aggregation [103].

**Figure 6:**
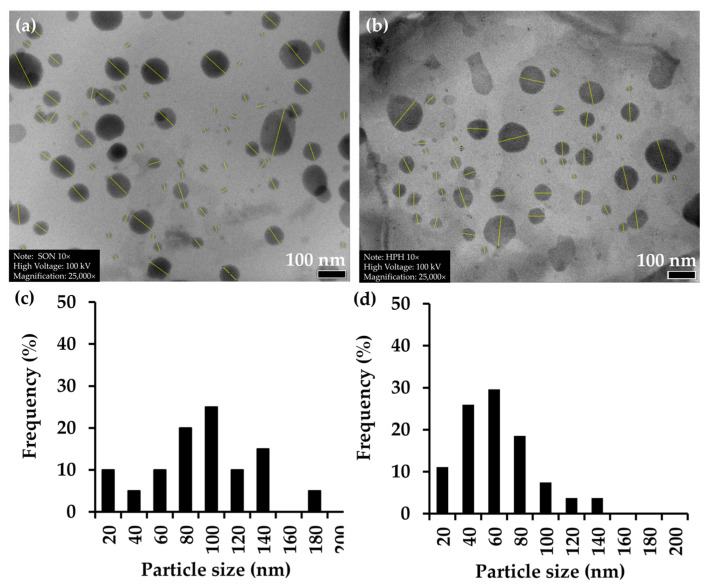
Transmission electron microscopy (TEM) images of *A. mellifera* brood fat extract-based nanostructured lipid carriers (NLCs) prepared by the probe sonication method are shown at scale bar of 100 nm (**a**), while NLCs prepared by the high-pressure homogenization method are shown at scale bar of 100 nm (**d**). Yellow lines indicate particle size measurements performed using ImageJ analysis. The corresponding particle size distribution histograms derived from the TEM images for samples (**a**) and (**b**) are shown in (**c**) and (**d**), respectively. The frequency (%) represents the relative number of particles within each size range.

Figure 6 presents representative micrographs of the NLC formulations prepared by probe sonication and high-pressure homogenization. As illustrated in Figure 6a, the formulation produced by probe sonication consisted predominantly of spherical to quasi-spherical nanoparticles with a relatively broader size distribution, including the presence of some larger particles. ImageJ analysis of the TEM images revealed particle sizes mainly ranging from approximately 20 to 180 nm, with the majority of particles distributed between 80 and 140 nm. The corresponding histogram (Figure 6c) confirms a relatively wide distribution, suggesting moderate polydispersity in the nanoparticle population. This observation is consistent with the higher mean particle size obtained from DLS measurements. In contrast, Figure 6b demonstrates that the NLCs prepared by high-pressure homogenization were more uniformly dispersed and exhibited a noticeably smaller and more homogeneous particle population, corroborating the reduced mean particle size as reported in Table 6. The TEM image analysis further confirmed these findings, as the particle sizes were mainly distributed between 20 and 120 nm, with the highest frequency occurring in the range of 40–80 nm, as illustrated in Figure 6d. For both preparation methods, the NLCs displayed smooth surfaces and well-defined boundaries, indicating the successful formation of stable lipid nanostructures without evident aggregation. The absence of extensive clustering in the TEM images, along with the relatively narrow particle size distribution shown in the histograms, further supports the low PDI values (0.13 to 0.14) and the good colloidal stability observed for both formulations. Therefore, the TEM findings were in good agreement with the physicochemical characterization results, confirming that high-pressure homogenization was more effective than probe sonication in producing smaller, more homogeneous NLCs with improved nanoscale organization.

**Figure 7:**
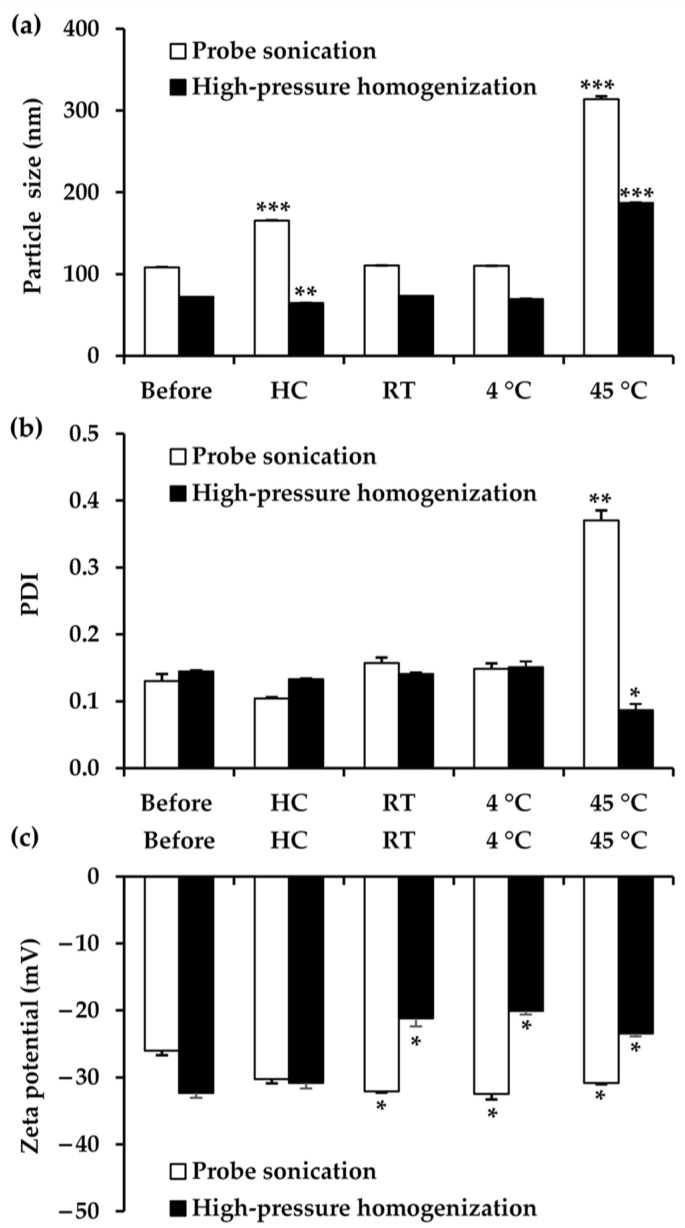
Particle size (**a**), polydispersity index (PDI) (**b**), and zeta potential (**c**) of *A. mellifera* brood fat extract-based NLCs prepared using probe sonication (◻) and high-pressure homogenization (■) methods. Measurements were taken before, after eight heating–cooling cycles (HC; each cycle: 24 h at 4 °C followed by 24 h at 45 °C), and after one month of storage at room temperature (RT), 4 °C, and 45 °C. Asterisks (*) indicate significant differences compared with initial values (Before) (* *p* < 0.05; ** *p* < 0.01; *** *p* < 0.001).

The stability of the selected NLC formulations prepared using probe sonication and high-pressure homogenization was evaluated under stress conditions and during one-month storage at various temperatures (see below). Both NLC formulations were generally physically stable, with particle size and PDI remaining within the acceptable range after eight heating–cooling cycles and one month of long-term storage at various temperatures [104], except at the high temperature of 45 °C, as shown in Figure 7. At this elevated temperature, both the particle size and PDI of the NLCs increased dramatically after one month, suggesting instability under these conditions. This phenomenon can be attributed to Ostwald ripening, in which larger particles grow at the expense of smaller ones, leading to an increased average particle size of the NLCs [105,106]. Despite a significant change in the surface charge of the EA extract based-NLC prepared by high-pressure homogenization after the stability test (from −32.33 to −21.20 at room temperature, from −32.33 to −20.07 at 4 °C, and from −32.33 to −23.43 at 45 °C), its particle size and PDI remained unchanged. This observation may be explained by the presence of non-ionic surfactants (Tween^®^ 80 and Span^®^ 80) in the formulations, which stabilize colloidal systems viasteric hindrance rather than electrostatic repulsion [107]. Unlike electrostatic stabilization, steric hindrance creates a physical barrier around the particles, preventing aggregation even when zeta potential is low. Therefore, the mean particle size, PDI, and zeta potential of the EA extract-based NLC prepared by probe sonication remained within the acceptable range throughout the one-month stability study, consistent with the homogeneous appearance of the formulation and the absence of phase separation or segregation. Comparatively, the EA extract-based NLC prepared by high-pressure homogenization showed smaller particle sizes and better thermal stability across most conditions, reflecting the efficiency of high-pressure homogenization in producing uniform, robust NLCs.

### 3.7. Irritation Properties and Cytotoxicity of NLC from A. mellifera Brood Fat Extracts

**Figure 8:**
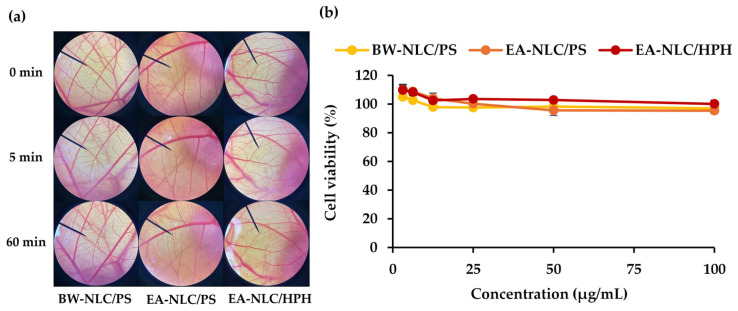
Chorioallantoic membrane (**a**) and cytotoxic effects on RAW 264.7 cells (**b**) of NLCs from commercial beeswax prepared using probe sonication (BW-NLC/PS), NLCs prepared from *A. mellifera* brood fat ethyl acetate extract using probe sonication (EA-NLC/PS), and NLCs prepared from *A. mellifera* brood fat ethyl acetate extract using high-pressure homogenization (EA-NLC/HPH).

The irritation properties and cytotoxicity of NLCs prepared from *A. mellifera* brood fat extracts were compared with those of beeswax-based NLCs, as shown in Figure 8. The findings indicated that the CAM after application of NLCs formulated with both beeswax and *A. mellifera* brood fat extracts showed no observable signs of vascular irritation or vessel damage, confirming their safety. In addition, cytotoxicity results demonstrated that all NLC formulations maintained high cell viability values above 90% across the entire investigated concentration ranges (up to 100 μg/mL). Although a slight decrease in cell viability was observed with increasing concentration, the reduction was minimal for all samples. Therefore, all NLC formulations induced no significant irritation in the CAM assay and exhibited no notable cytotoxic effects in vitro within the tested concentration range, indicating their suitability and biocompatibility for further topical applications [80].

### 3.8. Anti-Inflammatory Properties of NLC from A. mellifera Brood Fat Extracts

**Figure 9:**
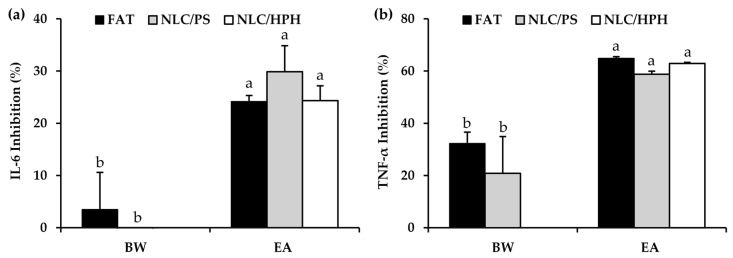
Inhibitory activities on IL-6 (**a**) and TNF-α (**b**) in RAW 264.7 cells stimulated with lipopolysaccharide (LPS) following treatment with beeswax (BW) and *A. mellifera* brood fat ethyl acetate extract (EA) in terms of native fat (FAT) or NLCs prepared using probe sonication (NLC/PS) and NLCs prepared using high-pressure homogenization (NLC/HPH). Superscript letters a and b indicate significant differences in inhibitory activities among treatments, as determined by one-way ANOVA with Tukey’s post hoc test (*p* < 0.05).

The inhibitory effects of NLC formulations prepared from *A. mellifera* brood fat extracts on the pro-inflammatory cytokines IL-6 and TNF-α, in comparison with commercial beeswax, are presented in Figure 9. In terms of IL-6 inhibition (Figure 9a), beeswax-based NLC formulations exhibited minimal inhibitory activity, showing no significant difference compared with native beeswax. Notably, their inhibitory activity was significantly lower than that of NLC formulations based on *A. mellifera* brood fat extracts (*p* < 0.05). Moreover, no significant difference was observed between the native *A. mellifera* brood fat extract and its NLC formulations prepared by either probe sonication or high-pressure homogenization. In contrast, EA extract-based formulations demonstrated markedly higher IL-6 inhibition, ranging from approximately 24.3 ± 2.8% to 29.9 ± 5.0%, which was significantly higher than that of beeswax-based NLC formulations. These results suggest that the intrinsic anti-inflammatory properties of the EA extract play a dominant role in IL-6 suppression, while NLC had no effect on their efficacy.

Similarly, TNF-α inhibition results (Figure 9b) revealed a clear distinction between beeswax and *A. mellifera* brood fat extract formulations. Beeswax exhibited lower TNF-α inhibition, with no significant differences between the beeswax alone and its NLC formulations (32.3 ± 4.3% and 20.8 ± 14.1, respectively). In contrast, EA extract-based NLC formulations demonstrated significantly higher TNF-α inhibition, reaching approximately 58.8 ± 1.2% to 64.9 ± 0.7% (*p* < 0.05). No significant differences in TNF-α inhibition were observed between the native EA extract and its NLC formulations, indicating that incorporation into NLCs did not significantly alter TNF-α inhibitory activity compared with the free fat extract. Therefore, it was obvious that EA extract possessed superior anti-inflammatory activity compared to beeswax, as evidenced by its stronger inhibition of both IL-6 and TNF-α (*p* < 0.05). NLC formulations maintained the anti-inflammatory effects without compromising activity, demonstrating that nanoencapsulation is a suitable strategy for delivering lipid-based bioactives. Importantly, the absence of significant differences between the native fats and NLC formulations suggests that the biological activity of the lipid extracts was preserved during the NLC preparation process. These findings, together with the favorable safety and cytotoxicity profiles, support the potential application of EA extract-based NLCs as safe and effective anti-inflammatory delivery systems.

## 4. Conclusions

This study demonstrated the potential of *A. mellifera* brood fat extracts as sustainable, bioactive lipid excipients and anti-inflammatory agents, offering a promising alternative to conventional beeswax. The extraction solvent significantly influenced lipid yield and composition, with EA emerging as the most suitable solvent due to its high extraction efficiency, favorable fatty acid profile, and greener solvent characteristics. Compared with beeswax, *A. mellifera* brood fat extracts exhibited markedly higher unsaturated fatty acid contents, particularly oleic acid, which contributed to their semisolid nature and superior biological activity. Physicochemical characterization revealed that *A. mellifera* brood fat extracts shared similar functional groups with beeswax while displaying lower crystallinity and distinct thermal degradation behavior, reflecting differences in lipid composition and molecular organization. All *A. mellifera* brood fat extracts were non-irritating in the HET-CAM assay and exhibited no significant cytotoxicity in RAW 264.7 macrophages, confirming their safety and biocompatibility for topical applications. *A. mellifera* brood fat extracts showed significantly greater anti-inflammatory activity than beeswax by more effectively inhibiting LPS-induced IL-6 and TNF-α production. The incorporation of EA extract into NLCs resulted in stable nanoscale formulations with favorable particle size, narrow polydispersity, and suitable zeta potential. High-pressure homogenization produced smaller and more uniform nanoparticles compared with probe sonication, highlighting its effectiveness for NLC preparation. Notably, the NLCs preserved the intrinsic anti-inflammatory activity of the *A. mellifera* brood fat extract without inducing irritation or cytotoxicity, indicating that the biological functionality of the lipid was maintained during formulation. The current study highlights *A. mellifera* brood fat extract, particularly EA extract, as a novel, safe, and effective lipid source for anti-inflammatory topical delivery systems and supported its potential application as a bioactive and sustainable alternative to beeswax in cosmetic and pharmaceutical formulations. These findings underpin the growing potential of insect-derived lipids as practical and versatile materials for the development of functional nanocarriers.

## Figures and Tables

**Table 1:** External appearance and yields of *A. mellifera* brood fat extracts.

*A. mellifera* Brood Fat Extracts	AC	EA	HX
External appearance	* 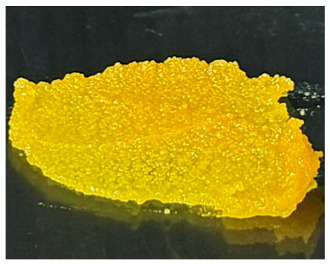 *	* 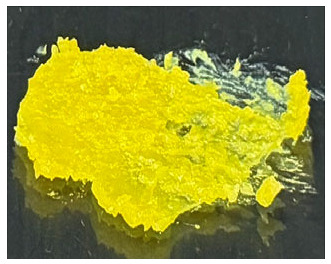 *	* 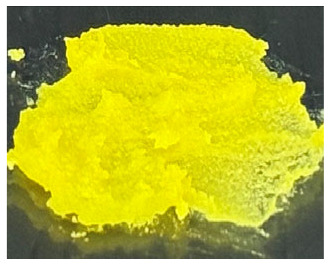 *
Yield (% *w*/*w*)	22.8 ± 0.0 ^b^	29.0 ± 1.0 ^a^	27.8 ± 0.4 ^a^

*A. mellifera* brood fat extracts were extracted using acetone (AC), ethyl acetate (EA), and hexane (HX). Superscript letters a and b indicate significant differences in yield between extracts determined by one-way ANOVA with Tukey’s post hoc test (*p* < 0.05).

**Table 2:** Fatty acid profile of *A. mellifera* brood fat extracts in comparison with beeswax.

Fatty Acid Composition	Concentration (% *w*/*w*)			
	BW	AC	EA	HX
Lauric acid (C12:0)	0.2 ± 0.0	0.2 ± 0.0	0.2 ± 0.0	0.2 ± 0.0
Myristic acid (C14:0)	0.4 ± 0.0 ^b^	3.0 ± 0.0 ^a^	3.0 ± 0.0 ^a^	3.0 ± 0.1 ^a^
Palmitic acid (C16:0)	47.9 ± 0.4 ^a^	42.6 ± 0.1 ^c^	43.8 ± 0.1 ^b^	43.9 ± 0.3 ^b^
Stearic acid (C18:0)	2.7 ± 0.0 ^c^	9.8 ± 0.1 ^b^	9.9 ± 0.1 ^ab^	10.1 ± 0.1 ^a^
Arachidic acid (C20:0)	0.3 ± 0.0	0.3 ± 0.0	0.3 ± 0.0	0.3 ± 0.0
Behenic acid (C22:0)	2.2 ± 0.0 ^a^	0.2 ± 0.0 ^b^	0.2 ± 0.0 ^b^	0.1 ± 0.0 ^c^
Lignoceric acid (C24:0)	19.8 ± 0.1 ^a^	1.9 ± 0.2 ^b^	1.9 ± 0.2 ^b^	1.0 ± 0.0 ^c^
Saturated fatty acids (SFA)	73.6 ± 0.3 ^a^	57.9 ± 0.2 ^c^	59.3 ± 0.1 ^b^	58.6 ± 0.4 ^bc^
Palmitoleic acid (C16:1)	3.9 ± 0.1 ^a^	0.7 ± 0.0 ^b^	0.7 ± 0.0 ^b^	0.7 ± 0.0 ^b^
Oleic acid (C18:1)	21.4 ± 0.2 ^c^	40.3 ± 0.2 ^a^	38.9 ± 0.1 ^b^	39.6 ± 0.4 ^ab^
cis-11-Eicosenoic acid (C20:1)	0.4 ± 0.0 ^a^	0.1 ± 0.0 ^b^	0.1 ± 0.0 ^b^	0.1 ± 0.0 ^b^
Erucic acid (C22:1)	0.0 ± 0.0 ^b^	0.2 ± 0.0 ^a^	0.2 ± 0.0 ^a^	0.2 ± 0.0 ^a^
Monounsaturated fatty acids (MUFA)	25.8 ± 0.3 ^c^	41.2 ± 0.2 ^a^	39.8 ± 0.1 ^b^	40.5 ± 0.4 ^ab^
Linoleic acid (C18:2)	0.5 ± 0.0 ^a^	0.3 ± 0.0 ^b^	0.3 ± 0.0 ^b^	0.3 ± 0.0 ^b^
Linolenic acid (C18:3)	0.1 ± 0.0 ^b^	0.6 ± 0.0 ^a^	0.6 ± 0.0 ^a^	0.6 ± 0.0 ^a^
Polyunsaturated fatty acids (PUFA)	0.6 ± 0.0 ^b^	0.9 ± 0.0 ^a^	0.9 ± 0.0 ^a^	0.9 ± 0.0 ^a^
Unsaturated fatty acid (USFA)	26.4 ± 0.3 ^c^	42.1 ± 0.2 ^a^	40.7 ± 0.1 ^b^	41.4 ± 0.4 ^ab^

BW = commercial beeswax; AC = acetone extract; EA = ethyl acetate extract; HX = hexane extract. Superscript letters a, b, and c indicate significant differences in fatty acid composition among BW and *A. mellifera* brood fat extracts determined by one-way ANOVA with Tukey’s post hoc test (*p* < 0.05).

**Table 3:** Comparison of FTIR spectral features of *A. mellifera* brood fat extracts and beeswax.

FTIR Spectral Features	*A. mellifera* Brood Fat Extracts	Beeswax [67]
CH_3_ asymmetric stretching	2960–2957 cm^−1^ (broad)	2957 cm^−1^
CH_2_ asymmetric & symmetric stretching	Asymmetric 2920 cm^−1^ Symmetric 2850 cm^−1^	Asymmetric 2922 cm^−1^ Symmetric 2852 cm^−1^
C=O stretching (ester)	1760 cm^−1^	1739 cm^−1^ (monoester)
CH_2_ bending	1465 cm^−1^ (scissor) 720 cm^−1^ (rocking)	1465 cm^−1^ (scissor) 720 cm^−1^ (rocking)
C=O stretching (free fatty acids)	1714 cm^−1^	1714 cm^−1^
C–O stretching/ ester vibrations	1172 cm^−1^	1172 cm^−1^
Fingerprint region	1500–1000 cm^−1^	1500–800 cm^−1^

*A. mellifera* brood fat extracts refer to *A. mellifera* brood fat extracts extracted using acetone (AC), ethyl acetate (EA), and hexane (HX).

**Table 4:** Irritation score and irritation potency of *A. mellifera* brood fat extracts.

Samples	Irritation Score	Irritation Potency
Positive control	17.7 ± 1.0 ^a^	Severe irritation
Negative control	0.0 ± 0.0 ^b^	No irritation
Vehicle control	0.0 ± 0.0 ^b^	No irritation
BW	0.0 ± 0.0 ^b^	No irritation
AC	0.0 ± 0.0 ^b^	No irritation
EA	0.0 ± 0.0 ^b^	No irritation
HX	0.0 ± 0.0 ^b^	No irritation

Positive control = 1% *w*/*v* of sodium lauryl sulfate aqueous solution; negative control = 0.9% *w*/*v* of sodium chloride (normal saline solution); vehicle control = isopropyl myristate (IPM; BW = 1% *w*/*v* commercial beeswax in IPM; AC = 1% *w*/*v* acetone extract in IPM; EA = 1% *w*/*v* ethyl acetate extract in IPM; HX = 1% *w*/*v* hexane extract in IPM). Superscript letters a and b indicate significant differences in fatty acid composition among BW and *A. mellifera* brood fat extracts determined by one-way ANOVA with Tukey’s post hoc test (*p* < 0.05).

**Table 5:** Particle size, polydispersity index (PDI), and zeta potential of NLCs prepared from beeswax and different *A. mellifera* brood fat extracts with various solid-to-liquid lipid ratios.

Solid-to-Liquid Lipid Ratio	BW	AC	EA	HX
	Particle size (nm)			
5:0	355.1 ± 2.0 ^b^	1642.7 ± 25.9 ^a^	178.5 ± 0.5 ^e^	282.3 ± 1.5 ^c^
3.5:1.5	299.3 ± 0.3 ^c^	154.2 ± 0.8 ^g^	108.0 ± 0.6 ^h^	171.0 ± 0.9 ^f^
2.5:2.5	227.1 ± 2.5 ^d^	83.9 ± 0.4 ^j^	82.1 ± 0.2 ^j^	96.0 ± 0.2 ^i^
1.5:3.5	93.7 ± 0.6 ^i^	72.3 ± 0.2 ^k^	76.9 ± 1.2 ^jk^	82.2 ± 0.2 ^j^
	PDI			
5:0	0.21 ± 0.02 ^d^	1.00 ± 0.00 ^a^	0.19 ± 0.01 ^de^	0.49 ± 0.01 ^b^
3.5:1.5	0.34 ± 0.02 ^c^	0.15 ± 0.01 ^eh^	0.13 ± 0.01 ^gh^	0.18 ± 0.00 ^def^
2.5:2.5	0.31 ± 0.03 ^c^	0.13 ± 0.00 ^gh^	0.15 ± 0.01 ^fh^	0.15 ± 0.00 ^fh^
1.5:3.5	0.14 ± 0.02 ^fh^	0.11 ± 0.01 ^h^	0.16 ± 0.02 ^efg^	0.16 ± 0.01 ^efg^
	Zeta potential (mV)			
5:0	−31.0 ± 0.8 ^ef^	−32.1 ± 0.6 ^fg^	−29.8 ± 0.4 ^de^	−28.9 ± 0.2 ^ce^
3.5:1.5	−28.2 ± 0.4 ^cd^	−27.9 ± 0.3 ^bcd^	−26.0 ± 0.7 ^ab^	−30.7 ± 0.4 ^ef^
2.5:2.5	−25.8 ± 0.3 ^a^	−36.7 ± 1.0 ^h^	−34.0 ± 1.4 ^g^	−32.8 ± 0.3 ^fg^
1.5:3.5	−25.2 ± 0.6 ^a^	−25.5 ± 0.8 ^a^	−27.2 ± 0.8 ^ac^	−30.7 ± 0.9 ^ef^

BW = commercial beeswax; AC = acetone extract; EA = ethyl acetate extract; HX = hexane extract; PDI = polydispersity index. Superscript letters a–k indicate significant differences in NLC characteristics among BW and *A. mellifera* brood fat extracts determined by one-way ANOVA with Tukey’s post hoc test (*p* < 0.05).

**Table 6:** Particle size, polydispersity index (PDI), and zeta potential of NLCs prepared from *A. mellifera* brood EA extracts at a solid-to-liquid lipid ratio of 3.5:1.5 using probe sonication and high-pressure homogenization.

Methods	Particle Size (nm)	PDI	Zeta Potential (mV)
Probe sonication	108.0 ± 0.6 ^b^	0.13 ± 0.01 ^a^	−26.0 ± 0.7 ^b^
High-pressure homogenizer	72.1 ± 0.3 ^a^	0.14 ± 0.00 ^b^	−32.3 ± 0.7 ^a^

NLCs were prepared with sugar squalane as the liquid lipid and a surfactant system consisting of 5% *w*/*w* Tween^®^ 80 and Span^®^ 80 (HLB = 11). Values are presented as mean ± SD (n = 3). PDI = polydispersity index. Superscript letters a and b indicate significant differences in NLC characteristics among probe sonication and high-pressure homogenization determined by one-way ANOVA with Tukey’s post hoc test (*p* < 0.05).

## Data Availability

The data supporting the findings of this study are included in this article.

## Supplementary Materials

The following supporting information can be downloaded at: https://www.mdpi.com/article/10.3390/biology15060472/s1, Figure S1: DSC thermograms of *Apis mellifera* brood fat extracts extracted using acetone (AC), ethyl acetate (EA), and hexane (HX).

## Abbreviations

The following abbreviations are used in this manuscript:

AC	Acetone extract
ANOVA	Analysis of variance
ATCC	American Type Culture Collection
BW	Beeswax
CAM	Chorioallantoic membrane
COX-2	Cyclooxygenase-2
DMEM	Dulbecco’s Modified Eagle Medium
DLS	Dynamic light scattering
DMSO	Dimethyl sulfoxide
DSC	Differential scanning calorimetry
DX	Dexamethasone
EA	Ethyl acetate extract
ELISA	Enzyme-linked immunosorbent assay
FBS	Fetal bovine serum
HC	Heating–cooling cycle
HET-CAM	Hen’s egg test–chorioallantoic membrane assay
HLB	Hydrophilic–lipophilic balance
HX	Hexane extract
IL-6	Interleukin-6
iNOS	Inducible nitric oxide synthase
IPM	Isopropyl myristate
IS	Irritation score
LPS	Lipopolysaccharide
MAPK	Mitogen-activated protein kinase
MTT	3-(4,5-Dimethylthiazol-2-yl)-2,5-diphenyltetrazolium bromide assay
NF-κB	Nuclear factor kappa B
NLC	Nanostructured lipid carrier
NLC/HPH	Nanostructured lipid carrier prepared by high-pressure homogenization
NLC/PS	Nanostructured lipid carrier prepared by probe sonication
NSS	Normal saline solution
PDI	Polydispersity index
PPARs	Peroxisome proliferator-activated receptors
RAW 264.7	Murine macrophage cell line
RT	Room temperature
SFA	Saturated fatty acids
SLN	Solid lipid nanoparticle
SLS	Sodium lauryl sulfate
TEM	Transmission electron microscopy
TGA	Thermogravimetric analysis
TNF-α	Tumor necrosis factor alpha
USFA	Unsaturated fatty acids
