# Supplementary material for: Brood-Derived Fat Extracts from Apis mellifera as Sustainable Alternatives to Beeswax in Topical Nanostructured Lipid Carriers
Source: Biology (Basel). 2026 Mar 14;15(6):472. doi: 10.3390/biology15060472 (PMC13023864; doi:10.3390/biology15060472)
Supplement: Supplementary file 1 [file biology-15-00472-s001.zip › biology-4180875-supplementary.pdf]

biology
MDPI

Supplementary data

Brood-Derived Fat Extracts from *Apis mellifera* as Sustainable Alternatives to Beeswax in Topical Nanostructured Lipid Carriers

Piyathida Samianpet, Suvimol Somwongin, Rewat Phongphisutthinant, Supakit Chaipoot, Pairote Wiriyacharee, Singkome Tima, Songyot Anuchapreeda, Saranya Juntrapirom, Watchara Kanjanakawinkul, Thomas Rades and Wantida Chaiyana*

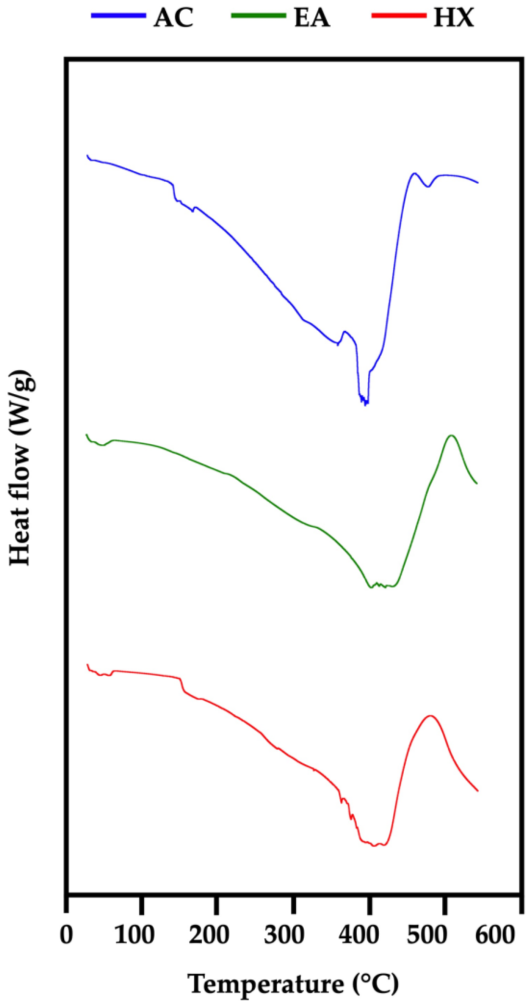

Figure S1: DSC thermograms of *Apis mellifera* brood fat extracts extracted using acetone (AC), ethyl acetate (EA), and hexane (HX).
